# Determination of A1 and A2 β-Casein in Milk Using Characteristic Thermolytic Peptides via Liquid Chromatography-Mass Spectrometry

**DOI:** 10.3390/molecules28135200

**Published:** 2023-07-04

**Authors:** Zeyang Liu, Susu Pan, Peize Wu, Ming Li, Dapeng Liang

**Affiliations:** 1 Key Laboratory of Groundwater Resources and Environment of the Ministry of Education, Jilin University, Changchun 130012, China; 2 Division of Chemical Metrology & Analytical Science, National Institute of Metrology, Beijing 100029, China; 3 Division of Ecology Environment and Energy Resources, Beijing Institute of Metrology, Beijing 100012, China

**Keywords:** β-casein, A2 milk, thermolysin, liquid chromatography, tandem mass spectrometry

## Abstract

β-casein, a protein in milk and dairy products, has two main variant forms termed as A1 and A2. A1 β-casein may have adverse effects on humans. The fact that there is only one amino acid variation at the 67th position between A1 and A2 β-casein makes it difficult to distinguish between them. In this study, a novel method using characteristic thermolytic peptides is developed for the determination of A1 and A2 β-casein in milk. Firstly, caseins extracted from milk samples are thermolytic digested at 60 °C without any denaturing reagents required for unfolding proteins, which simplifies the sample pretreatment procedure. The characteristic thermolytic peptides (i.e., fragments 66–76 and 59–76 for A1 and A2 β-casein, respectively) selected to specifically distinguish A1 and A2 β-casein only have eleven or eighteen amino acid moieties. Compared with tryptic characteristic peptides with a length of 49 amino acid moieties, these shorter thermolytic characteristic peptides are more suitable for LC-MS analysis. This novel method, with the advantages of high specificity, high sensitivity, and high efficiency, was successfully applied for the analysis of six milk samples collected from a local supermarket. After further investigation, it is found that this method would contribute to the development of A2 dairy products for a company and the quality inspection of A2 dairy products for a government.

## 1. Introduction

The ingestion of milk protein plays an indispensable role in human metabolism, nutrition acquisition and physical health [1]. About 80% of milk protein is casein [2], mainly including α, β and κ-casein, among which β-casein (β-CN) accounts for about 40% [3]. More than ten variant forms of β-CN exist in milk, and A1 β-CN and A2 β-CN are the two main forms [4]. The only difference between these two variants is the amino acid moiety at the 67th position, i.e., there is a proline moiety in A2 β-CN, while there is histidine moiety in A1 β-CN [5]. Although this difference is very slight, the bioactive peptides produced by the digestion of these two variants have significantly different effects on humans [6]. A1 β-CN, when digested, produces β-casomorphin-7 (BCM-7) [7], which can cause many diseases [8]. Conversely, BCM-7 is not generated from A2 β-CN [9]. In addition, BCM-7 is more easily absorbed by infants and young children compared with adults [10]. It was also found that the BCM-7 level in the urine of children with autism was significantly higher than that in healthy children, and the severity of illness was positively correlated with the BCM-7 level [11]. In many countries, milk and dairy product containing only A2 β-CN are labeled as A2 products for the consumer to make targeted choices. Therefore, the determination of the β-CN variant is of great significance for food manufacturers as well as consumers.

Originally, milk and dairy products with different β-CN variants were directly traced to milk collected from different cattle populations [12]. Several methods based on different principles have also been developed to determine different β-CN variants in milk or various dairy products. For example, an electrophoresis coupled with multiple linear regression (MLR) method could initially predict various caseins in cheese [13]. A urea–polyacrylamide electrophoresis method was developed to distinguish A1 and A2 β-CN variants in milk [14]. A polymerase chain reaction (PCR) combined with the high-resolution melting method has also been applied to identify β-CN variants [15,16]. In recent years, liquid chromatography–mass spectrometry with advantages of high sensitivity, high accuracy, and high specificity is widely used in various fields. Liquid chromatography has the capability of separating different proteins in milk, while high-resolution mass spectrometry has the advantages of high specificity and high sensitivity in determining the molecular weights of intact proteins. However, it is very difficult to completely separate intact β-CN variants using liquid chromatography because their physical and chemical properties are extremely similar. In addition, direct detection of the molecular weights for intact caseins [17] creates high requirements for the mass range and ionization performance of a mass spectrometer. Alternatively, it is more feasible to determine β-CN variants through detecting characteristic peptides after enzyme digestion [18]. Recently, characteristic peptides with a length of 49 amino acid moieties, produced by trypsin digestion, were reported to determine A1 and A2 β-CN in milk [19]. The molecular weights of these characteristic tryptic peptides are much higher than the ideal mass range (i.e., 700–1500 daltons) [20] for mass spectrometric analysis.

In this study, thermolysin was adopted for use in protein digestion. Considering that the difference between A1 and A2 β-CN variants is the amino acid moiety at 67th position in the sequence, the observed β-CN fragments 66–76 and 59–76 after digestion were selected as characteristic peptides for A1 and A2 β-CN, respectively. The characteristic thermolytic peptides of A1 and A2 β-CN were obviously different in both composition and length, which facilitates liquid chromatography separation. Meanwhile, the shorter characteristic peptides could be beneficial for mass detection and peptide standard synthesis [21]. A method based on liquid chromatography–mass spectrometry (LC-MS) has been established to determine characteristic thermolytic peptides of A1 and A2 β-CN variants in milk. These characteristic peptides were analyzed by means of MS/MS experiments under collision-induced dissociation (CID) mode, which further increases the specificity of the method, and thus the reliability of the result. For application demonstration, six commercial milk samples (i.e., four normal milk samples and two A2 milk samples) were analyzed using this method. The results prove that this method has the advantages of high specificity, high sensitivity, and high efficiency, and thus would contribute to the development of A2 dairy products for a company and the quality inspection of A2 dairy products for a government.

## 2. Results and Discussion

### 2.1. Choice of Thermolytic Characteristic Peptides

To fully dissolve the proteins, a buffer solution is usually prepared with urea, dithiothreitol, or inorganic salts added [22], which contributes to protein unfolding and thus improves the efficiency of extraction and enzymatic digestion. The sample should be desalted before being subjected to LC-MS analysis, avoiding contamination of the mass spectrometer. To improve extraction efficiency and reduce sample preparation time, a protein extraction method based on isoelectric precipitation was developed in this study. A Tris-HCl buffer solution was used to extract caseins. After sufficient precipitation, caseins were isolated by means of centrifugation at 4 °C. The low-temperature centrifugation can reduce the interaction between hydrophobic groups in the milk protein, preventing the aggregation of whey protein and casein.

Thermolysin preferentially cleaves the amide bond positioned at the N-terminus of the hydrophobic residues such as leucine, phenylalanine, valine, isoleucine, alanine, and methionine. Thermolysin still maintains activity at 60 °C [23], which facilitates protein unfolding without additional denaturing reagents being required, and it thus is conducive to digestion [24]. Therefore, thermolysin was chosen as a digestion enzyme in this study. Thermolytic fragments containing different amino acid moieties at the 67th position in β-caseins were selected as the characteristic peptides distinguishing A1 and A2 β-caseins, and the sequences of A1 peptide and A2 peptide are listed in Table 1.

**Figure 1:**
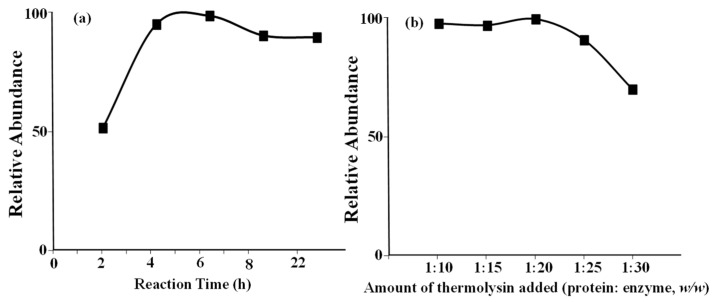
Optimization of experimental conditions. (**a**) Reaction time. (**b**) Amount of thermolysin added.

One of the favored cleavage sites is the N-terminus of position P1′, Ile66 in this case. However, thermolytic cleavage is blocked when proline is located at position P2′, as reported previously [25], e.g., Pro67 for A2 protein. This is the reason that different lengths of A1 and A2 peptides were observed. Similarly, cleavage of Ile74 was not blocked because of the presence of Pro75. Therefore, no other peptide in the Pro/His67 area was observed in this study. The digestion experimental conditions were carefully optimized using a β-casein standard. The relative abundances of A1 peptide, which was generated after enzyme digestion, against reaction time, and the amount of thermolysin are shown in Figure 1a,b, respectively. When the incubation time and amount of thermolysin added were optimized, the same trends for the A2 peptide level were also observed under the same condition. Finally, a digestion reaction time of 4 h and a protein to enzyme ratio of 1:20 were adopted for the experiments. Under optimized digestion conditions, no intact proteins were detected using the mass spectrometer, which proved that the reaction had been completed.

### 2.2. Chromatographic Separation and MS/MS Analysis

**Figure 2:**
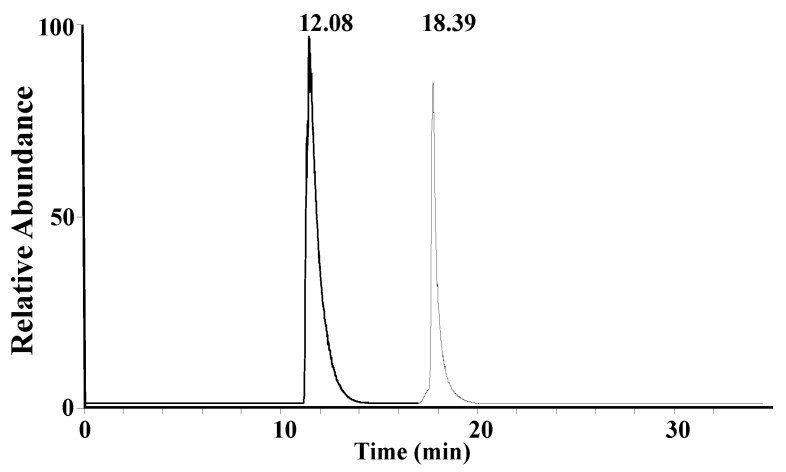
Overlap of extracted ion chromatograms of the A1 peptide and A2 peptide in a mixture standard solution (15 μg/mL).

A mixture solution of synthetic A1 peptide and A2 peptide standards (15 μg/mL) was used to optimize the liquid chromatography gradient. A core–shell LC column, which was previously found to be suitable for the separation of peptides [26,27], was chosen for the separation of β-CN digestion products. A1 peptide and A2 peptide can be completely separated, as shown in Figure 2.

**Figure 3:**
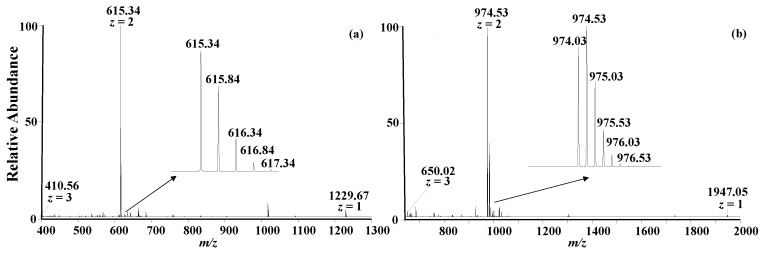
(**a**) Full mass spectrum of A1 peptide—the insert shows the isotope distribution. (**b**) Full mass spectrum of A2 peptide—the insert shows the isotope distribution.

A relatively long LC gradient of 30 min was adopted in this study, reducing the matrix effect as much as possible. When a shorter LC gradient is developed, analytical efficiency will be significantly increased, while slightly compromising the measurement accuracy. After liquid chromatographic separation, the peptides were analyzed using a high-resolution mass spectrometer and the full mass spectra of A1 peptide and A2 peptide are shown in Figure 3a,b, respectively.

In the full mass spectra, several multiple charge states were clearly observed as expected [28]. The most intense peaks correspond to the doubly charged species for A1 peptide (*m*/*z*: 615.34) and A2 peptide (*m*/*z*: 974.03). The isotope distributions exhibited in the inserts of Figure 3a,b are consistent with the theoretical data obtained from a program on a website (https://www.sisweb.com/mstools/isotope.htm, accessed on 16 June 2023). The relative errors of the exact masses are less than 6.3 ppm, as listed in Table 1. The experimental MS/MS spectra of the peptides upon collision-induced dissociation are also consistent with the theoretical data.

### 2.3. Method Validation

A soybean milk was chosen as a blank for the negative control study, no thermolytic characteristic peptides were detected after the sample preparation process. Thermolysis experiments were performed six times in six days, and the characteristic peptides (i.e., A1 β-CN fragment 66–76 and A2 β-CN fragment 59–76) were always observed. The relative standard deviations (*n* = 6) of reproducibility were calculated to be 3.1% and 2.9% for A1 peptide and A2 peptide, respectively. Several methods using isotope-labelled peptides or heavy-labelled peptides are available for accurate protein quantification after enzyme digestion. This study mainly aims to report novel characteristic thermolytic peptides for the determination of A1 and A2 β-casein in milk. Therefore, the standard addition method, which is relatively simple, was performed for quantitative analysis of characteristic peptides in the thermolytic digestion solution. After a series of A1 peptide and A2 peptide stock solutions were added, the total amounts of A1 peptide and A2 peptide in the digestion solution were measured by using the established LC-MS^2^ method. A calibration curve for measuring A1 peptide was obtained with a satisfactory linearity (R^2^ = 0.998), and acceptable relative standard deviations (RSD) of 3.6~4.5% (*n* = 6). A calibration curve for measuring A2 peptide was obtained with a satisfactory linearity (R^2^ = 0.992) and relative standard deviations (RSD) of 3.2~4.8% (*n* = 6). Consequently, the original concentrations of A1 peptide and A2 peptide were calculated based on the linear responds against total peptide concentrations. Finally, the amounts of A1 protein and A2 protein in the milk samples were calculated, assuming that they were equal to the amounts of A1 peptide and A2 peptide, respectively. The limits of detection (LOD, S/N = 3) for A1 peptide and A2 peptide were determined to be 25 ng/mL, and 31 ng/mL, respectively. Therefore, LODs for A1 protein and A2 protein in the digestion sample were calculated to be 0.48 μg/mL, and 0.38 μg/mL, respectively. With the dilution steps during sample preparation considered, the LODs for A1 protein and A2 protein in a milk sample were calculated to be 0.22 mg/mL and 0.18 mg/mL, respectively. Notably, the LODs for A1 protein and A2 protein in a milk sample could be significantly improved by slightly reducing the dilution fold during the sample preparation process.

### 2.4. Determination of A1 and A2 β-CN in Milk

For the application demonstration, six commercial milk samples (i.e., four normal milk samples and four A2 milk samples) collected from a local supermarket were analyzed using this novel method. For the negative control, a soybean milk sample was analyzed using the same method.

**Figure 4:**
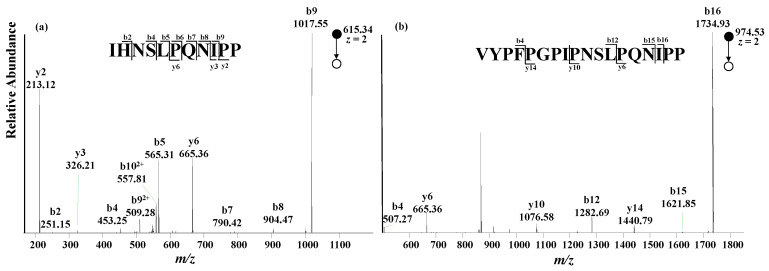
MS/MS spectra of A1 peptide (**a**) and A2 peptide (**b**) in the milk samples analyzed.

With an LC-MS method, A1 and A2 peptides could be determined based on their retention times and exact masses, distinguishing β-CN variants. However, false signals could be generated if an isobaric peptide with the same retention time is present in the digestion solution sample. Alternatively, tandem mass spectrometry can further increase the specificity of the LC-MS method, and thus improve the reliability of the result. In this study, the characteristic peptides containing varied amino acid moieties at the 67th position were subjected to fragmentation upon collision-induced dissociation. The fragmental ions were clearly annotated, as shown in Figure 4, and are consistent with the theoretical MS/MS data.

An extracted ion chromatogram constructed from the tandem mass spectrum, instead of the full mass spectrum, could avoid the possible false signals from the isobaric peptides with the same retention time as characteristic peptides, which greatly increases the specificity and sensitivity of the analytical method. Given that the fragmental ions annotated as b_9_ for A1 peptide and b_16_ for A2 peptide (shown in Figure 4) are the two most abundant product ions, b_9_(A1) and b_16_(A2) were chosen to quantitatively analyze A1 peptide and A2 peptide. Meanwhile, the fragmental ions y_6_ (A1) and y_6_ (A2) were chosen to assess peptide identity, increasing method specificity. A standard addition method was adopted for quantitative analysis by adding A1 and A2 peptide mixed standard solutions in the thermolytic digestion solution. The mass spectrometer was set to the MS^2^ mode with a collision energy of 18 arb. Integrated areas of b_9_ (A1) and b_16_ (A2) peaks in the extracted ion chromatogram from the tandem mass spectrum were used as quantitative signals for establishing standard addition curves. As listed in Table 2, both A1 peptide and A2 peptide were detected for all four normal milk samples. The A1 β-CN level is approximately two to four times higher than the A2 β-CN level in the normal milk samples analyzed.

For A2 milk sample 1, an A2 peptide signal exclusively appeared in the overlay of EIC for A1 and A2 peptides, as expected. For A2 milk sample 2, an A1 peptide signal with small intensity also appeared in the overlay of EIC for A1 and A2 peptides. Compared with the four normal milk samples, the A1 peptide signal in A2 milk sample 2 is significantly lower than the A2 peptide signal, indicating that only a small amount of the A1 β-CN variant exists in A2 milk sample 2. Although the manufacturer states that the A2 milk product exclusively contains A2 β-CN variant, the existence of a very small amount of A1 β-CN variant still can be detected using the characteristic thermolytic peptides based on LC-MS/MS.

## 3. Experimental Section

### 3.1. Reagents

Triton X-100, β-casein standard from bovine milk, and thermolysin were purchased from Sigma-Aldrich (St. Louis, MO, USA). Sodium chloride was purchased from Suprapur (Merck, Darmstadt, Germany). Tris(hydroxymethyl) aminomethane was purchased from GPC Biotechnology (Beijing, China). Formic acid was purchased from Fluka (Buchs, Switzerland). Ultrapure water (18.2 MΩ·cm^−1^) was produced using a Milli-Q system (Fisher Scientific, San Jose, CA, USA). The peptide standards of A1 and A2 β-CN fragments 66–76 and 59–76 (named as A1 peptide, A2 peptide, respectively) were synthetized by SBS (Beijing, China). Six commercial milk samples including four normal milk samples and two A2 milk samples were purchased from a local supermarket.

### 3.2. Instrument

A high-speed refrigerated centrifuge (CR 21GⅢ, Hitachi, Hitachinaka, Japan), pH meter (FE20-K, Mettler Toledo, Zurich, Switzerland) and orbital incubator (SI500, Stuart, Stone, UK) were used for sample pretreatment. A series 1250 liquid chromatograph coupled with a linear quadrupole ion trap (LTQ)-Orbitrap Velos Pro mass spectrometer (Thermo Fisher Scientific, San Jose, CA, USA) was used for peptide separation and detection. An electrospray ionization source (ESI) was used for ion generation. The mass spectrometer was operated in positive ion mode. Collision-induced dissociation (CID) in an ion trap was adopted for tandem mass spectrometry (MS/MS) experiments.

### 3.3. LC Parameters

Liquid chromatographic separation was performed using a core–shell LC column (Kinetex C18, 150 × 4.6 mm, 2.6 μm, 100 Å; Phenomenex, Torrance, CA, USA). The injection volume was set at 10 μL, and the flow rate was set at 0.3 mL/min. Eluent A consisted of water:formic acid (1000:1, *v*/*v*) and eluent B consisted of acetonitrile:formic acid (1000:1, *v*/*v*). The LC mobile phase gradient was started at 5% eluent B, then gradually increased to 55% eluent B in 25 min, and then gradually increased to 95% eluent B in 10 min.

### 3.4. MS Parameters

To obtain optimal signal intensities for the peptides, four parameters (source voltage, sheath gas flow rate, auxiliary gas, and capillary temperature) were carefully optimized. The optimized MS parameters were set as: ESI needle position D (vertical), 2 (forward–backward); source voltage, 3.5 kV; sheath gas flow rate, 5 arb (arbitrary units); auxiliary gas flow rate, 20 arb for A1 peptide, 5 arb for A2 peptide; capillary temperature, 300 °C. The mass resolution of the Orbitrap was set at 30,000. The data type was set to “profile”, and the mass range was set at *m*/*z* 150–2000 Th. For MS/MS experiments, the normalized collision energy was set at 18 arb for A1 peptide and 21 for A2 peptide under CID mode.

### 3.5. Sample Preparation

Caseins in milk were isolated by means of isoelectric precipitation from crude mixtures. Firstly, 10 mL of the milk sample was dissolved in 40 mL of tris-HCl solution (50 mmol/L). Then, 10 mL of the abovementioned solution and 10 mL of a NaCl solution (0.5 mol/L) were mixed using a vortex mixer. The pH of this mixture solution was adjusted to 4.6–4.8 with a 10% formic acid aqueous solution. The sample solution was stored at 4 °C in a freezer overnight, waiting for caseins to be completely precipitated. Then, the sample was centrifugated for 15 min (4 °C, 10,000 r/min). The precipitate collected was dissolved in 10 mL of tris-HCl solution (50 mmol/L). Then, 500 μL of the casein solution mentioned above was diluted two-fold with ultrapure water, and a suitable amount of thermolysin (1 mg/mL) was added to perform an enzymatic digestion reaction at 60 °C for 4 h. The digestion reaction was terminated by adding 20 μL of a 10% formic acid aqueous solution. The digestion product was centrifuged for 10 min (4 °C, 10,000 r/min) and the supernatant was collected for subsequent LC-MS analysis.

### 3.6. Standard Addition Method

In the standard addition method, 0 μL, 5 μL, 10 μL, 15 μL, and 20 μL of A1 and A2 peptide mixed standard solution (15 μg/mL) were separately spiked into five 250 μL digestion samples. Then, ultrapure water was added into the spiked samples, reaching a constant volume of 500 μL. Finally, contents of A1 peptide and A2 peptide in the abovementioned samples were measured by using the established LC-MS^2^ method. Extracted ion chromatogram was constructed from a tandem mass spectrum using b_9_ (A1) and b_16_ (A2). Areas of b_9_ (A1) and b_16_ (A2) peaks in the extracted ion chromatogram from the tandem mass spectrum were manually integrated to establish standard addition curves. The x-intercept of the extrapolated line was calculated as the original concentration of the analyte in the sample solution. Given that the digestion sample was diluted twice during the standard addition process, the contraction of the analyte in the original digestion sample can be obtained by multiplying it by a factor of 2.

### 3.7. Data Evaluation

All the mass spectra were recorded using Xcalibur^®^ software (version 2.2) equipped in the instrument (Thermo Fisher Scientific, San Jose, CA, USA). Theoretical data of MS/MS product ions of peptides produced by enzymatic digestion were calculated using the MS-Product tool available from a website (https://prospector2.ucsf.edu/prospector/cgi-bin/msform.cgi?form=msproduct, accessed on 16 June 2023). The possible sequences of peptides generated from thermolytic digestion of β-casein were predicted using the Peptide-Cutter tool available from a website (https://web.expasy.org/peptide_cutter/, accessed on 16 June 2023).

## 4. Conclusions

In this study, a novel method has been developed to determine A1 and A2 β-CN variants in milk using characteristic thermolytic peptides. Casein extraction from milk samples was achieved by means of isoelectric precipitation with a tris-HCl buffer solution at pH 4.6–4.8. Thermolysin, chosen as an enzyme for casein digestion, maintains activity at a higher temperature of 60 °C, which facilitates protein unfolding and thus improves the efficiency of enzymatic digestion. After thermolytic digestion, characteristic peptides containing the varied amino acid moiety at the 67th position in the β-CN sequence were selected as signatures determining A1 and A2 β-CN variants in milk samples. Compared with tryptic characteristic peptides with a length of 49 amino acid moieties, these thermolytic characteristic peptides (i.e., fragments 66–76 and 59–76 for A1 and A2 β-CN, respectively) are relative shorter, which are more suitable for LC-MS analysis. Under the optimized liquid chromatography gradient, A1 peptide and A2 peptide could be separated completely. With the information regarding the retention times, molecular weights obtained from full mass spectra, and fragmental behaviors of characteristic peptides from tandem mass spectra, unambiguous determination of β-CN variants can be successfully accomplished. Finally, six commercially available milk samples including four normal milk samples and two A2 milk samples were analyzed for the application demonstration of this novel method. The A1 β-CN variant in all of the normal milk samples tested accounts for the majority. No A1 β-CN variant was detected in one A2 labeled milk sample, and a small amount of an A1 β-CN variant was clearly detected in another A2-labeled milk sample. Although the amount of this A1 β-CN variant in this A2-labeled milk product is very low, it may still cause adverse effects in humans. To reduce the potential risk to customers, it would be very helpful to develop a method to determine A1 and A2 β-casein in milk.

This novel method developed here has the advantages of high sensitivity, high specificity, and high efficiency. After further investigation, it would contribute to the development of A2 dairy products for a company and the quality inspection of A2 dairy products for a government.

## Figures and Tables

**Table 1:** Detailed information of A1 peptide and A2 peptide.

Peptide	Amino Acid Sequence	Theoretical Mass (Da) [M + H]^+^	Observed Mass (Da) [M + H]^+^	Error (ppm)
A1	IHNSLPQNIPP	1229.664	1229.671	6.3
A2	VYPFPGPIPNSLPQNIPP	1947.038	1947.049	6.1

**Table 2:** Contents of A1 β-casein and A2 β-casein detected in 6 commercially available milk samples (mg/mL).

Milk Sample	A1 β-Casein	A2 β-Casein
Normal 1	5.98	3.13
Normal 2	6.81	1.98
Normal 3	8.68	2.62
Normal 4	5.59	1.51
A2 sample 1	<0.22	4.87
A2 sample 2	0.66	3.15

## Data Availability

The data that support the findings of this study will be available from the corresponding authors upon reasonable request.

## Sample Availability

Samples of the compounds are not available from the authors.
